# What’s happening where when SARS-CoV-2 infects: are TLR7 and MAFB sufficient to explain patient vulnerability?

**DOI:** 10.1186/s12979-022-00262-3

**Published:** 2022-01-22

**Authors:** Ludwig Englmeier, Julien Subburayalu

**Affiliations:** 1 Center for Regenerative Therapies Dresden (CRTD), Technische Universität Dresden, Fetscherstrasse 105, 01307 Dresden, Germany; 2 Patent Attorney Dr. Ludwig Englmeier, scrIPtum, Erlenaustrasse 11, 83080 Oberaudorf, Germany; 3 Mildred Scheel Early Career Center, Medical Faculty, Technische Universität Dresden, Dresden, Germany; 4 Center for Regenerative Therapies, Technische Universität Dresden, Dresden, Germany

**Keywords:** COVID-19, SARS-CoV-2, Obesity, Age, TLR-tolerance, TLR7, MAFB, miR-146a, Macrophages, Plasmacytoid dendritic cells

## Abstract

The present COVID-19 pandemic has revealed that several characteristics render patients especially prone to developing severe COVID-19 disease, i.e., the male sex, obesity, and old age. An explanation for the observed pattern of vulnerability has been proposed which is based on the concept of low sensitivity of the TLR7-signaling pathway at the time of infection as a common denominator of vulnerable patient groups.

We will discuss whether the concept of established TLR-tolerance in macrophages and dendritic cells of the obese and elderly prior to infection can explain not only the vulnerability of these two demographic groups towards development of a severe infection with SARS-CoV-2, but also the observed cytokine response in these vulnerable patients, which is skewed towards pro-inflammatory cytokines with a missing interferon signature.

## Introduction

In the present COVID-19 pandemic several patient characteristics have emerged to increase the risk for developing severe COVID-19 disease, i.e., the male sex, obesity, and old age (reviewed in Gao et al., 2021) [1]. A theory has been proposed which is based on the concept of low sensitivity of the toll-like receptor (TLR)7-signaling pathway in macrophages and plasmacytoid dendritic cells (pDCs) at the time of infection as a common denominator of vulnerable patient groups. These immune cells of the ’innate’ lineage are endowed with an arsenal of pattern recognition receptors (PRRs), including TLRs, thereby steadily patrolling tissues and the circulation to orchestrate the host’s immune response to microbial agents and confer tissue protection [2, 3].

TLR7 escapes X-chromosomal inactivation in women, who thus have a higher expression of TLR7 than men. This could explain the lower sensitivity of the TLR7-signaling pathway in men due to lower gene dosage [4]. In the obese and elderly the establishment of TLR-tolerance prior to infection could explain the lower sensitivity of TLR7-signaling [5]. This was the model which was previously suggested to account for the observed risk profile of the three demographic groups [6].

Mechanistically, the chronic activation of TLRs prior to infection in the obese and elderly would lead to the development of TLR-tolerance [7, 8]. A key player in the establishment of this TLR-tolerance is microRNA (miR)-146a. Chronic engagement of the TLR-pathway induces high levels of miR-146a [9], directly resulting in a lower sensitivity of TLR7-signaling as visible in the transcriptional signatures of TLR-tolerant cells [10]. Enhanced levels of miR-146a coinciding with a reduced sensitivity to TLR7-signaling have been observed, for example, in peritoneal macrophages of old mice [11]. miR-146a downregulates the mRNA levels of components of the TLR7-signaling pathway, interleukin (IL) 1 receptor associated kinase 1 (IRAK1) and TNF-receptor associated factor 6 (TRAF6), at the post-transcriptional level (reviewed in Nahid et al., 2011) [12], providing a sound explanation for its role in TLR-tolerance.

If a viral infection occurred in such a condition of established TLR-tolerance, the initial interferon (IFN) response by macrophages and pDCs to the infectious agent could be expected to be blunted. Consequentially, this may facilitate systemic spread, enhance viral load, and confer vulnerability to the development of severe COVID-19 disease. Thus, the theory predicted that defects in TLR7-signaling would confer an increased degree of vulnerability toward infection with SARS-CoV-2.

A second prediction of the model was that after establishment of an infection with a single-stranded RNA virus such as SARS-CoV-2 in at-risk patients, the presence of the virus would at one point generate a strong enough danger signal to trigger a host immune response. Mechanistically, TLR-tolerance would be overcome in sentinel immune cells and sensitivity to TLR7-signaling restored, e.g., by down-regulation of miR-146a levels. Regained sensitivity to the TLR-signaling pathways would restore some functionality required to cope with the infection. However, this restored TLR-sensitivity comes at a cost for at-risk patients at a later stage of the infection due to then overactive TLR-signaling pathways, not only stimulated by viral RNA, but also by the intrinsic TLR substrates which had caused the initial TLR-tolerance in the first place.

### Predictions versus reality

One the one hand, this „desensitization/resensitization“ model predicted a particular role of TLR7 for determining sensitivity to infection with SARS-CoV-2. On the other hand, it predicted the emergence of systemic lupus erythematosus (SLE)-like biomarkers in severely ill COVID-19 patients due to an overactive TLR7-signaling pathway in a later phase of the disease. Since March 2020, when the initial theory had been developed, several results were published which are in accordance with this theorem.

### The importance of TLR7-signaling

Van der Made et al. (2020) have identified two sets of male siblings (median age of 26) characterized by a rare TLR7-loss-of-function-variant who suffered from severe COVID-19 disease [13]. This observation was confirmed by several other studies [14–16]. For example, Fallerini et al. reported five cases of men (three under 50 years, and two in their mid-60 s) with severe COVID-19 disease who carried rare TLR7-loss-of-function-variants.

Based on these publications van de Veerdonk and Netea (2021) concluded that variants of this single gene TLR7 are responsible for an important proportion of risk factor for severe COVID-19 in men under 50 due to the mutations leading to a loss-of-function in the antiviral response to SARS-CoV-2 [17].

Hence, the first part of the theory which proposed the development of severe infection with SARS-CoV-2 due to an insufficient immune response by sentinel immune cells of the ‘innate’ lineage as a consequence of dysfunctional TLR7-signaling at infection is now backed up by important supporting observational data.

### Severe COVID-19 disease and the similarity to systemic lupus erythematosus

The second part of the theory, i.e., the prediction that overactive TLR-signaling at later stages of COVID-19 disease would result in symptoms mimicking a TLR7-driven SLE-flare, is now also supported by ample observational evidence. Lupus anticoagulant has been found in 91% of a cohort of patients with severe COVID-19 disease [18]. Moreover, from a random cohort of severely ill COVID-19 patients 68.7% were tested positive for systemic autoantibodies, such as antinuclear antibodies (ANAs), which comprise a major determinant of the SLICC criteria for diagnosing SLE [19]. The role of hyperresponsive TLR7-signaling as one reason for the development of these autoantibodies in SLE patients is known in the field, and thus the appearance of these autoantibodies in patients suffering from severe COVID-19 disease implies overactive TLR7-signaling at a later stage, when the disease has progressed to a severe form.

**Fig. 1:**
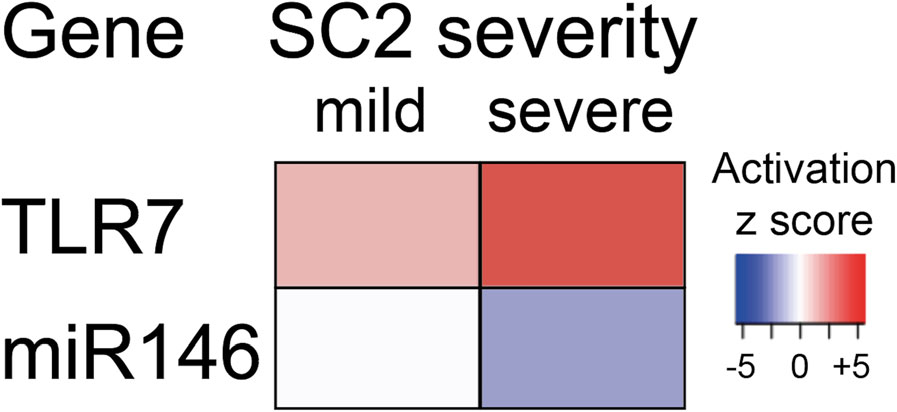
Shaath et al. (2020) have reported canonical pathways and upstream regulator networks associated with either mild or severe cases of SARS-CoV-2 (SC2). Bronchoalveolar samples from patients enabled a comparative transcriptomic analysis which tied an enhanced TLR7-expression to patients suffering from severe infection with SARS-CoV-2, whereas expression of the canonical miR146 was repressed in such patients compared to a mild severity of COVID-19 disease. For more genes differentially expressed refer to Shaath et al. (2020) [20]

Evidence at the level of gene expression is also accumulating that supports an activation of TLR7 signaling in patients with severe COVID-19 disease. Gene signatures obtained from single cell sequencing of bronchoalveolar lavage specimens from patients with severe COVID-19 disease were compared to those of healthy patients [20]. Interestingly, there is an upregulation of TLR7-transcripts in patients with severe disease coinciding with a downregulation of miR-146 [Fig. 1].

This offers a direct mechanistical basis for reactivated TLR7-signaling in patients with severe COVID-19 disease. We interpret this data by assuming that the observed upregulation of TLR7 and downregulation of miR-146 occur after infection in these patients because a strong early TLR7-mediated IFN response would have protected them from the development of severe disease [21]. This observation provides a rationale for how sensitivity to TLR7-signaling is restored after the infection with SARS-CoV-2 has established itself.

### What is happening where upon infection?

There are, however, several questions that the initial simple model did not answer, and two observations that it failed to explain.

The first question is, why the observed defects in TLR7-signaling should be so detrimental in the case of SARS-CoV-2, when there are redundant signaling pathways for viral pathogen-associated molecular patterns (PAMPs)? Secondly, assuming that TLR7-signaling is restored in the latter phase of infection, why should reengaged signaling via TLR7 result in the observed pattern of the cytokine storm, which is skewed towards pro-inflammatory cytokines and chemokines with a systemic IFN response missing [22]?

At a first glance, the dependence on strong signaling via the TLR7-pathway in the early phase of infection with SARS-CoV-2 might be seen as surprising, since there are two major pathways for the activation of an antiviral type I IFN response to infection with RNA viruses: RIG-I-like receptors (RLRs) and the Toll-like receptors (TLRs), respectively [23]. However, it has been reported that viral proteins from SARS-CoV-2 interfere with the RIG-I pathway. For example, Chen et al. (2020) have shown that SARS-CoV-2’s N protein represses IFN production by interfering with RIG-I [24]. Besides, its M protein antagonizes type I and Ill IFN responses by targeting both RIG-I and MDA-5 signaling alike [25]. In other words, the inhibition of the RLR pathway, a sensor of viral RNA in the cytoplasm, might offer a clue as to why the initial innate immune response is very dependent on functional TLR7-signaling, an endosomal sensor for viral RNA, in the case of infection with SARS-CoV-2.

Physiologically, viral infection of the respiratory tract brings about a dual kinetic of type I and type III IFN responses. As such, infected epithelial cells usually release type I and type III IFNs, which confer the expression of IFN-stimulated genes (ISGs) in neighboring cells to establish an antiviral environment [26]. These ISG signatures activate surveying sentinel immune cells and, therefore, confer host protection, also against SARS-CoV-2 [27, 28]. Patrolling macrophages and pDCs, unlike other hematopoietic cells, are highly responsive to type III IFNs [29, 30]. In turn, this set of ISGs fosters the production of a second wave of type I and type III IFNs by tissue macrophages and pDCs [31]. This antiviral response is key to further recruitment of monocytes and to enhance their maturation to myeloid DCs, ultimately augmenting their cross-priming activities to orchestrate natural killer cell and cytotoxic T cell activity during the early immune response [32–36].

In the case of SARS-CoV-2, however, entry of SARS-CoV-2 into epithelial cells of the nasopharyngeal tract or tracheobronchial system prevents the localized first peak of IFN response by infected epithelial cells owing to RIG-I/MDA-5 evasion. As a consequence, the infected epithelial cells cannot trigger the expression of ISGs in neighboring cells, and an antiviral environment is not established. With the IFN response of the epithelial cells missing, the IFN response by the host now completely relies on functional viral sensing mediated by TLR7 in sentinel immune cells underneath the epithelium. If this residual IFN response was abrogated because of genetic defects as described in young men with dysfunctional TLR7-variants or because of established TLR-tolerance in the elderly and obese, severe infection with SARS-CoV-2 would not be preventable, resulting in potentially debilitating and life-threatening consequences [13–15].

A model emerges where upon viral entry into epithelial cells at the site of first infection, localized IFN production by the infected cells is prevented by viral proteins like the nucleocapsid protein and/or the M-protein [24, 25], which inhibit signaling of the cytoplasmic RLRs. An early protective type I and type III IFN response by the host then relies on functional endosomal TLR7-mediated sensing of viral RNA by sentinel immune cells beneath the epithelium.

While SARS-CoV-2 enters plasmacytoid dendritic cells, which do not express angiotensin-converting enzyme 2 (ACE2), by a Neuropilin-1-dependent mechanism, it is not able to replicate in pDCs or macrophages. This observation could be explained by a model, where different receptors for viral entry, such as ACE2 and Neuropilin-1, deliver the virus to different subcellular compartments. In the case of ACE2 (or a combination of ACE2 and Neuropilin-1), the SARS-CoV-2 RNA reaches the cytoplasm and starts replicating [37]. However, if viral entry is mediated by Neuropilin-1 only, the virus is taken up into the endosomal compartment but never reaches the cytoplasm and thus never starts replicating. This would explain that pDCs and macrophages are able to sense the viral RNA (via endosomal TLR7), but are not permissive to virus replication. Recently, it was shown that the presence of viral RNA within the endosome alone is indeed insufficient to establish productive infection [38].

**Fig. 2:**
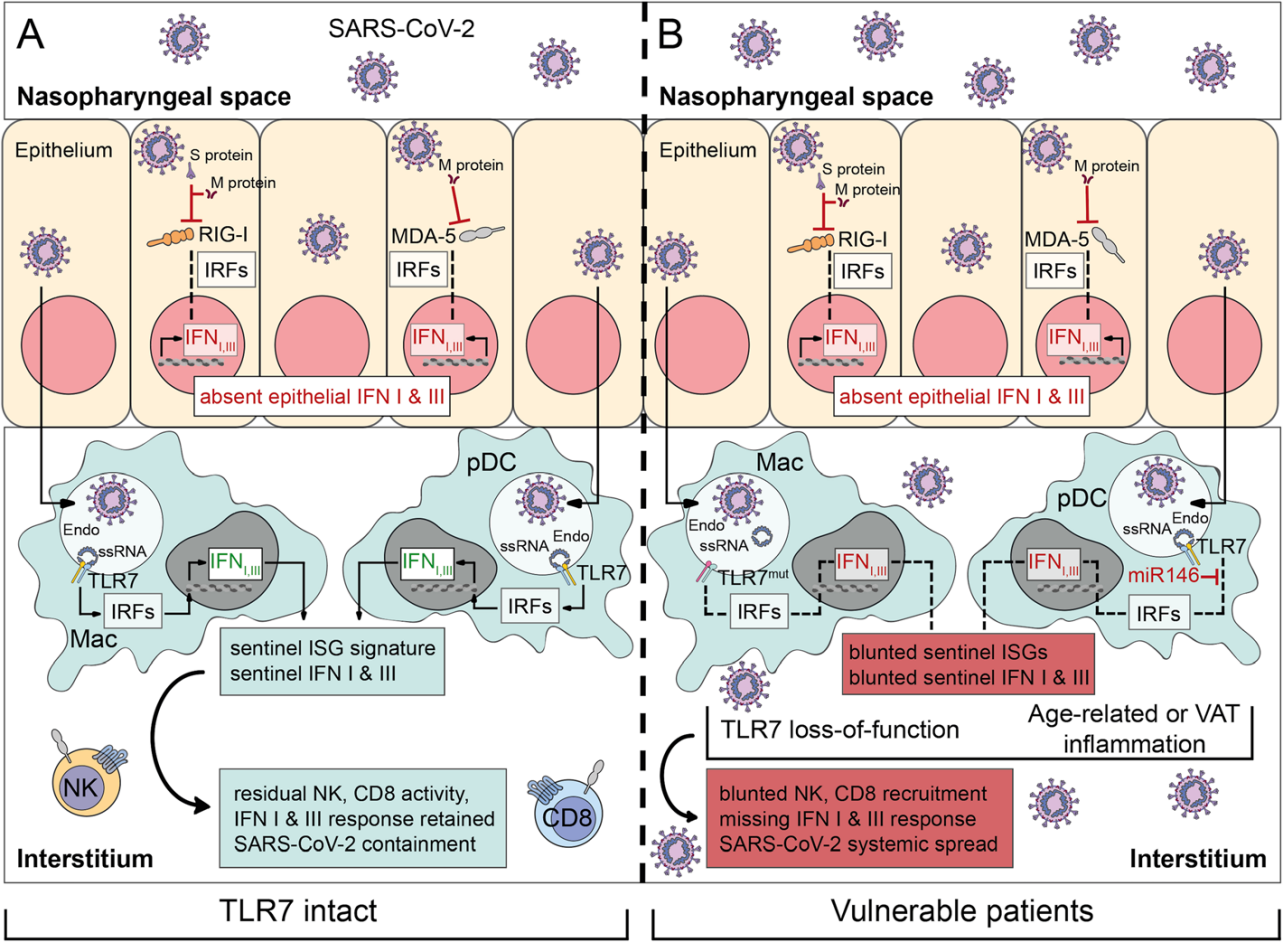
SARS-CoV-2 infects epithelial cells of the nasopharyngeal tract and enters the cytoplasm. Here, the Spike protein and the M-protein inhibit cytoplasmic pathogen-recognition receptors (PRRs) like RIG-I and MDA-5. This results in abrogated stimulation of interferon (IFN) regulatory factors (IRFs), which are key to eliciting an antiviral type I and type III IFN response. Below the infected epithelium sentinel immune cells like macrophages (Mac) or plasmacytoid dendritic cells (pDCs) encounter the virus by endosomal uptake. In the endosome, binding of single stranded RNA of SARS-CoV-2 results in activation of TLR7. In the case of an active toll-like receptor (TLR)7-signaling pathway (left), TLR7-engagement results in a cytokine and interferon response. This secondary IFN release by sentinel cells can bring about the antiviral response by augmenting natural killer (NK) and CD8 cytotoxic T cell activities, thereby containing infection with SARS-CoV-2, and preventing systemic spread. In contrast, vulnerable patients with either loss-of-function alleles of TLR7 or a downregulated TLR-signaling pathway prior to infection (right) lack also this second IFN response. Age-related inflammation or inflammation related to visceral adipose tissue (VAT) leads to an enhanced expression of microRNA-146a (miR-146). In turn, miR-146 targets downstream signaling molecules of the TLR7-pathway including interleukin-1 receptor-associated kinase 1 (IRAK1) or TNF receptor associated factor 6 (TRAF6). Ultimately, the response of the TLR7-signaling pathway is subdued even though the receptor TLR7 itself is sensing viral RNA, and the IFN response by sentinel cells is missing. SARS-CoV-2 can then spread systemically, thereby establishing severe forms of COVID-19 disease

Thus, because viral particles are endocytosed by sentinel immune cells [39], inhibitory viral proteins are not located in the cytoplasm but rather in the endosome, where they cannot interfere with the cytoplasmic downstream components of the TLR7-pathway. Therefore, SARS-CoV-2 does not interfere with this second type of IFN response. If, however, this second response was missing, e.g., due to genetic defects, as described in the young and healthy, but TLR7-mutant men identified by van der Made et al. or blunted in case of established TLR-tolerance of peripheral macrophages and dendritic cells in other vulnerable patient groups, severe infection by SARS-CoV-2 is facilitated. This concept is illustrated below [Fig. 2].

### The role of MAFB in the skewed cytokine response

Turning to the second question – and assuming that resensitization of the TLR7-signaling pathway does occur sometime after infection - why is the systemic response skewed, with an IFN response missing in the blood plasma [22]? Should a strong engagement of the TLR7-signaling pathway not result in both, pro-inflammatory cytokine secretion via nuclear factor kappa B (NF-κB) and an IFN response via IFN regulatory factor (IRF)7 (for a review see Kawai and Akira, 2010) [40]? Several recent publications allow an explanation for this discrepancy based on the concept of TLR-tolerance.

**Fig. 3:**
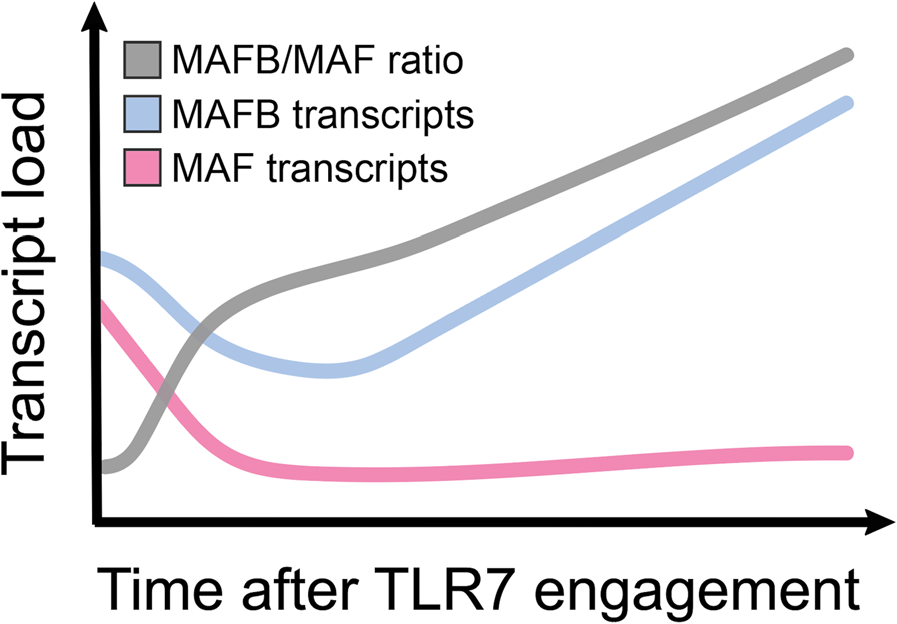
Vega et al. (2020) have exposed human monocyte-derived macrophages to the TLR ligand CL264 and have measured the transcriptional regulation of the transcription factors MAFB and MAF within hours following exposure. Stimulation with CL264 induced an increase in the transcript load for MAFB, whereas MAF mRNA levels were decreased, thereby increasing the MAFB/MAF ratio in TLR7-stimulated monocyte-derived macrophages

MAFB is a transcriptional antagonist of the IFN response, and functions by impairing the interaction of co-activators with IRFs [41]. Recently, it was suggested that the MAFB/MAF ratio determines the transcriptional profile of TLR7-activated macrophages [42]. Interestingly, this group also reported an increased expression of MAFB upon TLR7-engagement [42], summarized in Fig. 3. They suggest that in severe disease an increase in TLR7-signaling would lead to increased MAFB expression over time and consequently to the development of a skewed inflammatory response.

While the connection between TLR7, MAFB, and the IFN response offers an attractive possibility to explain the observed cytokine response, a model where increased MAFB expression because of increased engagement of TLR7 after viral infection raises several issues. At the time of infection, expression of MAFB in subepithelial sentinel cells should not be particularly high and thus those macrophages and pDCs should respond with IFN secretion immediately after viral infection of the epithelium Why should this response not be sufficient to contain infection with SARS-CoV-2 [21]? Why should the skewed IFN response develop selectively in at-risk patients if stimulation of TLR7 by viral RNA and the resulting induction of MAFB expression will happen in all patients? Why are young men with mutant TLR7 especially vulnerable [13–15]? And why should an increased expression of MAFB in macrophages be relevant for the missing systemic IFN response at all, which is mainly driven by pDCs and not by macrophages [43]?

### TLR-tolerance and its effect on the IFN response

**Fig. 4:**
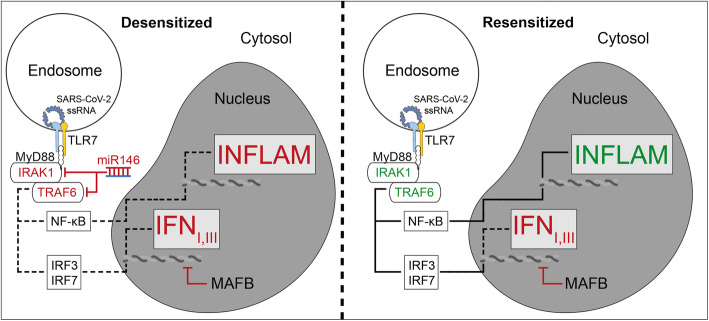
Resensitization of the TLR7-signaling pathway leads to a skewed cytokine response with a missing IFN response due to repression of IFN expression by high levels of the BZIP transcription factor MAFB, a known antagonist of antiviral responses

If, however, a low-level stimulation of TLR7 was present at steady state in at-risk patient groups, i.e., already prior to viral infection, the obese and elderly would be characterized by sentinel immune cells which are permissive to infection with SARS-CoV-2 because of endogenously high levels of miR-146a and detectable levels of MAFB. This model could explain the observed vulnerability of at-risk patients, the missing IFN response, and the development of the observed skewed cytokine response characteristic for severe COVID-19 disease. The expected situation in TLR-tolerant macrophages and pDCs in the desensitized state at the time of infection and the resensitized state during severe COVID-19 disease are illustrated below [Fig. 4]. This model provides a mechanistic rationale for the observed immune responses in the obese and elderly.

But is there a role for MAFB in pDCs at all? To date, the literature on MAFB is mainly based on observations in macrophages. However, Saiga et al. (2021) have recently reported that MAFB is expressed in pDCs, where it interacts with the Ets domain of Spi-B [44]. In pDCs, it disrupts the complex between IRF7 and Spi-B, thereby abrogating a type I IFN response in pDCs [44]. Conversely, inhibition of MAFB mRNA in the acute phase of an immune response led to an enhanced type I IFN response by pDCs [44].

### Is there a physiological role for TLR-tolerance?

There is still considerable scarcity regarding *in vivo* evidence for TLR-tolerance in macrophages and pDCs during homeostasis and its physiological consequences on the control of infection. However, the liver is an interesting example, since it is continuously exposed to the TLR4 agonist lipopolysaccharide (LPS) derived from the gut microbiome [45]. Indeed, LPS derived from the gut microbiome confers a TLR-tolerogenic state on liver DCs with broad effects not exclusively on TLR4 [45–47]. Due to this tolerogenic state, the liver is also more prone to tumor metastases and microbial infection than other organs [48, 49]. And because of TLR-tolerance, liver DCs behave also very differently to non-tolerant DCs of the peripheral blood [50]. Whereas DCs from the peripheral blood produce inflammatory cytokines in response to an engagement of TLRs, TLR-tolerant liver DCs produce IL-10 [50]. A similar TLR-tolerant state of macrophages and dendritic cells locating beneath infected epithelial cells would, therefore, lead to a situation mimicking the liver’s vulnerability toward microbial infection.

There might also be a beneficial role for TLR-tolerance in the physiological response to a virus infection in healthy young patients. In this patient group subepithelial macrophages are, as discussed, initially not TLR-tolerant and do provide an interferon-response upon first contact with the virus. At a later stage of the infection, however, a mechanism that drives macrophages towards an anti-inflammatory, M2-like polarization state upon continuous TLR7-stimulation via upregulation of MAFB, as described by Vega et al. [42] and Kim [51], should be beneficial. Macrophages are not specialized in the selective and coordinated removal of virus-infected cells, and rather coordinate the antiviral response by further recruitment of NK cells and cytotoxic T cells to the site of infection. When those specialist-cells arrive and start sending large numbers of virus-infected cells into apoptosis, the presence of anti-inflammatory macrophages, which have a higher capacity for phagocytosis and tissue remodeling than pro-inflammatory macrophages, will be useful for the disposal of apoptotic and necrotic cells and beneficial for the resolution of inflammation.

## Conclusions

We suggest the following addition to the initial “TLR-desensitization/resensitization” model [6] to explain the sensitivity of the obese and the elderly toward an infection with SARS-CoV-2:

- In the obese and elderly prior to infection, miR-146a and MAFB levels are high in pDCs and macrophages, which are in a TLR-tolerant state due to continuous low-level stimulation of TLRs.
- Upon infection, epithelial cells fail to generate a local type I and type III IFN response due to viral inhibition of cytoplasmic RIG-I/MDA-5 signaling.
- The immediate systemic type I and type III IFN response by endosomal TLR7 of the pDCs fails due to the TLR-tolerant state of pDCs in at-risk patients.
- After systemic viral infection is established, sensitivity to TLR-signaling is regained because of degradation or decreased expression of miR-146a in sentinel immune cells.
- However, expression of MAFB does not decrease at this crucial stage, or with delayed kinetics as compared to miR-146a. Instead, active TLR7-signaling further promotes or maintains expression of MAFB.
- pDCs with low levels of miR-146a and high levels of MAFB produce a skewed response, with strong NF-kB-signaling but blunted activation of IRFs. This results in the observed cytokine pattern in severely affected patients suffering from COVID-19 disease.

The observations by Shaath et al. (2020) [20], who have reported a decrease in mRNA levels of miR-146a and an increase in TLR7 in their single cell sequencing analysis, provide a mechanistic framework that is consistent with the aforementioned concept of desensitization and resensitization of the TLR7-signaling pathway in vivo. And in combination with the role of MAFB in the IFN response this would explain the strong but skewed innate immune response.

This model also predicts that the response of pDCs in a preparation of peripheral blood mononuclear cells to TLR7-agonists like imiquimod or R848 prior to infection could serve as an assay for predicting patient sensitivity.

## Data Availability

Not applicable.

## Abbreviations

ACE2: angiotensin-converting enzyme 2
IFN: interferon
IL: interleukin
IRAK1: interleukin 1 receptor associated kinase 1
IRFs: IFN regulatory factors
ISGs: IFN-stimulated genes
miR: microRNA
mRNA: messenger RNA
NF-κB: nuclear factor kappa B
NK: natural killer
PAMPs: pathogen-associated molecular patterns
pDC: plasmacytoid dendritic cells
PRRs: pattern recognition receptors
RLRs: RIG-I-like receptors
RNA: ribonucleic acid
SLE: systemic lupus erythematosus
TLRs: toll-like receptors
TNF: tumor necrosis factor
TRAF6: TNF receptor associated factor 6
VAT: visceral adipose tissue
